# High resolution physical mapping of single gene fragments on pachytene chromosome 4 and 7 of *Rosa*

**DOI:** 10.1186/s12863-015-0233-9

**Published:** 2015-07-02

**Authors:** Ilya V. Kirov, Katrijn Van Laere, Ludmila I. Khrustaleva

**Affiliations:** Department of Genetics, Biotechnology, Plant Breeding and Seed Science, Russian State Agrarian University - Moscow Timiryazev Agricultural Academy, Timiryazevskay str.49, 127550 Moscow, Russia; Center of Molecular Biotechnology, Russian State Agrarian University - Moscow Timiryazev Agricultural Academy, Listvennichnaya Alleya 5, 127550 Moscow, Russia; Plant Sciences Unit, Applied Genetics and Breeding, Institute for Agricultural and Fisheries Research (ILVO), Caritasstraat 21, 9090 Melle, Belgium

**Keywords:** Fluorescence In Situ Hybridization, Pachytene, Tyramide-FISH, *Rosa*, Physical map

## Abstract

**Background:**

Rosaceae is a family containing many economically important fruit and ornamental species. Although fluorescence in situ hybridization (FISH)-based physical mapping of plant genomes is a valuable tool for map-based cloning, comparative genomics and evolutionary studies, no studies using high resolution physical mapping have been performed in this family. Previously we proved that physical mapping of single-copy genes as small as 1.1 kb is possible on mitotic metaphase chromosomes of *Rosa wichurana* using Tyramide-FISH. In this study we aimed to further improve the physical map of *Rosa wichurana* by applying high resolution FISH to pachytene chromosomes.

**Results:**

Using high resolution Tyramide-FISH and multicolor Tyramide-FISH, 7 genes (1.7–3 kb) were successfully mapped on pachytene chromosomes 4 and 7 of *Rosa wichurana*. Additionally, by using multicolor Tyramide-FISH three closely located genes were simultaneously visualized on chromosome 7. A detailed map of heterochromatine/euchromatine patterns of chromosome 4 and 7 was developed with indication of the physical position of these 7 genes. Comparison of the gene order between *Rosa wichurana* and *Fragaria vesca* revealed a poor collinearity for chromosome 7, but a perfect collinearity for chromosome 4.

**Conclusions:**

High resolution physical mapping of short probes on pachytene chromosomes of *Rosa wichurana* was successfully performed for the first time. Application of Tyramide-FISH on pachytene chromosomes allowed the mapping resolution to be increased up to 20 times compared to mitotic metaphase chromosomes. High resolution Tyramide-FISH and multicolor Tyramide-FISH might become useful tools for further physical mapping of single-copy genes and for the integration of physical and genetic maps of *Rosa wichurana* and other members of the Rosaceae.

**Electronic supplementary material:**

The online version of this article (doi:10.1186/s12863-015-0233-9) contains supplementary material, which is available to authorized users.

## Background

*Rosa* is a genus of the Rosaceae family consisting of approximately 90 genera and approximately 3000 species. Many of these are economically important such as *Malus*, *Prunus*, *Pyrus*, *Fragaria*, *Rubus*, *Sorbus*, *Cotoneaster* and *Crataegus* [1–5]. Approximately 150 species and more than 20.000 cultivars of *Rosa* are described [6]. Most species have a complex origin [7]. Interestingly, only 7 to 15 species have contributed to the original germplasm of the modern rose cultivars [8]. *Rosa* species have small genomes and a high level of heterozygosity. Basic chromosome number is *x* = 7 [1], but ploidy levels range from diploid (2n = 2x = 14) to decaploid (2n = 8x = 56). Genomes of *Pyrus* [9], *Prunus* [10], *Fragaria* [4] and *Malus* [11] recently have been sequenced, providing valuable information for comparative genomics, gene cloning, marker development, QTL mapping and marker-assisted selection. Comparative analysis of these sequenced genomes has shed more light on the mode of evolution of some Rosaceae genera and species. In contrast, the organization of the *Rosa* genome has only been poorly investigated and the knowledge about the macro-synteny and collinearity of the Rose genome with other sequenced genomes Rosaceae family is poor.

Genetic maps have been widely used for comparative genomic and genome organization studies [12, 13]. The distance between markers in genetic maps expressed in recombination frequencies, or centimorgans (cM) is known to be unequally distributed along the chromosomes [14–17]. Gene order in genome regions with extremely low recombination frequency (e.g. centromeres, heterochromatin) cannot be revealed because of the low resolution of genetic mapping in these regions [18]. In addition, lack of collinearity between parental genomes used for development of the mapping population can cause inaccuracy in genetic maps [19]. In contrast to genetic maps, physical maps show real positions of DNA sequences on the chromosomes. Physical mapping using fluorescence in situ hybridization (FISH) does not depend on recombination frequency, therefore it can be used for gene mapping in “cold spot recombination” regions [18]. But FISH mapping has a lower efficiency than genetic mapping. The integration of physical and genetic maps provides a unique tool combining advantages of both types of maps. FISH-based physical maps have been developed and successfully integrated with genetic maps for many plant species (see review [20]). Direct visualization of DNA sequences on chromosomes by FISH is also a valuable for genome sequencing. FISH mapping improves the quality of genome assembly as demonstrated in tomato [21], cucumber [22] and Amborella [23].

Until now most of the cytogenetic studies in *Rosa* have been dedicated to karyotyping, chromosome number evaluation and rRNA (5S and 45S) gene mapping [24–28]. Further progress in FISH using individual genes or DNA clones (e.g. ESTs, BACs) is required for efficient cytogenetic map construction. Physical mapping of individual genes as small as 1.1 kb has proven to be possible on mitotic metaphase chromosomes of *Rosa wichurana* using Tyramide-FISH [29]. However, the resolution of Tyramide-FISH on the small mitotic *Rosa* chromosomes is very low which significantly hampers the construction of a physical map and the determination of the order of DNA sequences. The use of pachytene chromosomes would be an improvement for physical mapping [30]. Pachytene chromosomes are 7–40 times longer than mitotic metaphase chromosomes and therefore provide a higher resolution [21, 30]. Moreover, heterochromatic and euchromatic regions are distinguishable at the pachytene stage [31, 32]. Pachytene bivalents consist of 8 DNA strands instead of 4 in mitotic chromosomes, which also increases the sensitivity of FISH. Also important is that meiotic cells (pollen mother cells, or PMC) synchronously divide providing many cells in the same stage. High resolution FISH mapping on pachytene chromosomes has been used successfully in tomato [14, 21, 33–35] and *Arabidopsis* [31, 36–39]. However, for many plant species, pachytene preparation is still very challenging [30, 32, 40, 41].

*Rosa wichurana* is a diploid species (2n = 2x = 14) that was involved in the origin of modern rose cultivars. This species is a valuable source for resistance to powdery mildew [42, 43] and has been used for construction of several genetic maps [42–46]. Moreover, *R.wichurana* is attractive object for molecular cytogenetic studies because it has intensively growing apical meristems and simple corolla, providing many anthers to be used for pollen mother cells (PMC) isolation and pachytene chromosome preparation. Therefore *Rosa wichurana* is a good model for the *Rosa* genus,

In this paper, we improved the SteamDrop protocol [47] for preparation of high quality pachytene chromosomes of *Rosa wichurana*. We performed physical mapping of 7 genes on pachytene chromosomes 4 and 7 of *Rosa wichurana* using a high resolution Tyramide-FISH and multicolour Tyramide-FISH. For the first time, multicolor Tyramide-FISH was applied to plant chromosomes. The protocol for multicolor-FISH allowed simultaneous visualization of the physical positions of three genes closely located on chromosome 7 of *Rosa wichurana*.

## Methods

### An orthology-based approach for probe design resulted in FISH probes with a length between 1.7 kb and 3 kb

To isolate gene sequences on specific chromosomes of *R. wichurana* we used orthologous sequences of *Fragaria vesca* as a reference. The *Rosa wichurana* chromosomes 4 and 7 correspond to *Fragaria vesca* pseudochromosomes 7 (FvChr7) and 6 (FvChr6), respectively [29, 48]. Several genes from *Fragaria vesca* pseudochromosomes 6 and 7 (FvChr 6 and FvChr 7) were randomly selected using the NCBI Map Viewer tool. These candidate genes were then used for BLASTN analysis against the genome of *F. vesca* (FraVesHawaii_1.0) in order to estimate their copy number in the *Fragaria* genome and to select only the single-copy genes. Seven genes [MLO-like proteins (MLO2 and MLO3), ATPase (AAA-2), Ubiquitin protein ligase (RIN-2), monodehydroascorbate reductase (MDAR), Villin-2-like, mannosylglycoprotein endo-beta-mannosidase (MGM)] which showed significant similarity only to the original sequences of *Fragaria vesca* were chosen for further analysis. The selected genes were used for BLASTN against nucleotide and EST databases of *Rosa* at NCBI. Full mRNA sequences of *Rosa* (*Rosa multiflora*) MLO genes (JX847132.1, JX847133) were used for primer design to isolate MLO-like genes from *R.wichurana*. Pairwise alignment of rose MLO mRNA sequences with orthologous *Fragaria* full length MLO sequences was performed to prevent primer annealing at the intron/exon border.

The other 5 *Fragaria* full-length gene sequences were used for BLASTN against transcriptome reads of *R*. x hybrid (NCBI accession number: SRX097578) or *R. chinensis* transcriptome reads or/and clusters (https://iant.toulouse.inra.fr/). In this way, rose reads with significant similarity to different parts of the *Fragaria* genes were found and used for primer design.

### Amplification and labeling of gene fragments

Primers were designed using CLCbio Genomics Workbench version 7.0 to amplify gene fragments with a length more than 2 kb based on known *Fragaria* gene sequences and their location on the pseudochromosomes (Table 1). PCR products were generated using designed primers and genomic DNA of *R.wichurana*. PCR products were obtained with all 7 primer combinations. PCR with primers for *villin* resulted in a short PCR product of 900 bp, which is too short to be used in Tyramide-FISH experiments on *Rosa*. The other PCR products which ranged between 1.7 kb and 3 kb were cloned (Table 1) by pGEM-T easy (Promega, Madison, WI, USA) or by pPCR-TOPO kit (Invitrogen, Carlsbad, CA, USA). At least two clones were sequenced for each primer combination. The partial sequences of the clones used in this study are available as Additional file 1.

**Table 1:** Sequences of primers for gene fragment isolation and PCR results

Gene	Abbreviation	Primers, 5′-3′	Location on *Fragaria* pseudo-chromosomes	Expected length PCR product (bp)	Length obtained PCR products (bp)
MLO-like protein	MLO3	F: AAAACACCAACATGGGCAGT	FvChr 7: 15397809..15393519	1675	1700
		R: TTCCGAAAATCAAAGGTCGT			
MLO-like protein	MLO2	F: AGGATTTCAAGGTCGTGGTG	FvChr6: 34503533..34507423	1852	1800
		R: TGGTCGGCTAGCATTTTTCT			
ATPase	AAA-2	F: GTTCCCTTTGTCATTGCAG	FvChr7: 21485846.. 21481090	2718	3400
		R: ACGGCCTCTTCATCAATT			
Ubiquitin protein ligase	RIN-2	F: TCCTTCAGCTACACCATTGAC	FvChr7: 19866961..19871497	2228	2500
		R: AAATTGCGCGTTCCTACT			
Monodehydroascorbate reductase	MDAR	F: GAGGCGGTATGGTTAATTT	FvChr6: 12864594..12867898	2417	2800
		R: AAACTTGGGCTTTGGTGA			
Villin-2-like	Villin	F: CTCGCTTCTTCACAACATACT	FvChr6: 33309900..33321407	3851	900
		R:TTCACTGCCATTTTCATCCT			
Mannosylglycoprotein endo-beta-mannosidase	MGM	F: CGGCATGGAAAATGAGTCAA	FvChr6: 5180627..5186123	3017	3000
		R: GAACAAAGGGATCTGCCA			
Phenylalanine ammonia lyase^a^	PAL	F: ACCACTGGKTTTGGTGCWAC	FvChr6:34874086–34877587	-	1700
		R: CCYTTGAASCCATAATCCAA			
Pyrroline-5-Carboxylate Synthase^a^	P5CS	F: GCTGGCATCCCTGTTGTTAT	FvChr7: 17624431–17630820	-	1700
		R: CTTCGGATCGCTAATGAAGC			

^a^Data from [29]

For physical mapping of PAL and P5CS genes, previously cloned gene fragments [29], were used.

Plasmid DNA was isolated by the PureLink Quick Plasmid Miniprep Kit (Invitrogen, CA, USA) and labeled by Biotin-Nick translation mix (Roche, Mannheim, Germany), Digoxigenin-Nick Translation mix (Roche) or by a “home-made” Biotin-Nick translation Mix using a “home-labeled” dUTP nucleotide. “Home labeled” nucleotides were prepared according to the previously described protocol [49] using aminoallyl-dUTP (Thermo Scientific) and Biotinamidohexanoic acid N-hydroxysuccinimide ester (Sigma-Aldish Co., LLC, France). Our “home-made” Nick translation mix (30 μl) consists of 500 ng DNA, 83 μM dATP, dGTP, dCTP, 10.6 μM dTTP, 69 μM biotin-dUTP, 300 mU rDNAse I (Ambion) and 5U DNA Polymerase I (Invitrogen) in NT buffer (50 mM Tris–HCl, pH 7.8, 5 mM MgCl_2_) containing 10 mM beta-mercaptoethanol. The reaction was done at 15 °C for 2 h and stopped by heating at 80 °C for 5 min.

Clone pTa71 containing a 9-kb *Eco*RI fragment of the 45S rDNA from wheat [50], was labeled using the Digoxigenin-Nick Translation Mix (Roche).

### Pachytene chromosome preparation

*Rosa wichurana* plants were grown in a glass greenhouse under natural conditions in the temperate climate of East Flanders, Belgium. Five to ten anthers from each flower bud were placed on the slide and a drop of 60 % acetic acid was added. Anthers were then disrupted by a needle, heated at 55 °C for 1 min and squashed with a coverslip. The meiotic stages were observed under phase contrast (Zeiss AxioImager M2, Zeiss Company, Germany). Flower buds with most of the anthers containing PMCs in pachytene stage were fixed in Carnoy’s solution (3:1, ethanol:acetic acid) for at least 3 h and transferred into 70 % ethanol for storage at −20 °C.

Flower buds were washed in water for 30–40 min and transferred into 10 mM citric buffer (citric acid, sodium citrate dehydrate, pH 4.7–4.8) for 15–30 min. Anthers were then separated using a pincet and transferred into 50 μl enzyme mixture. One-third to one-fourth of all anthers from a single flower bud were used for the preparation of one tube of cell suspension. The enzyme mixture contained 0.6 % Pectolyase Y-23 (Kikkoman, Tokyo, Japan), 0.6 % Cellulase Onozuka R-10 (Yakult Co. Ltd., Tokyo, Japan) and 0.2 % Cytohelicase (Sigma-Aldish Co. LLC, France). A modified SteamDrop protocol [47] was used for cell suspension and chromosome preparation. Briefly, 10 μl of cell suspension were added on the slide; after 8–10 s, a drop of 22–28 μl of 1:1 ethanol:acetic acid was applied. When the slide surface became granule-like (50–80 s) steam (3–5 s) was applied to the slides and a second drop of 15–18 μl of 1:2 ethanol:acetic was added. When the granule-like surface appeared again on the slide, steam was applied and 300 μl of 45 % or 60 % acetic acid was added. Then slides were incubated on a heating plate (42 °C) for 15–30 s. A needle was used to spread the drop of acetic acid over the ring of cells on the slide. Acetic acid was removed by positioning the slide vertically on filter paper.

### Tyramide-FISH

We used the Tyramide-FISH protocol with an indirect detection system previously optimized for *R. wichurana* mitotic chromosomes [29]. Prehybridization procedures for single color and two color Tyramide-FISH included overnight incubation at 37 °C, 4 % paraformaldehyde treatment (6–7 min) and dehydration in an ethanol series (70 %, 90 % and 100 %). Probe hybridization and detection for single color Tyramide-FISH were carried out as described [29]. For two color Tyramide-FISH probe hybridization was conducted according to Kirov et al. [29] with a hybridization mixture containing two labeled probes: digoxigenin labeled MLO2 gene and biotin labeled PAL gene. The two probes were detected sequentially. First, the PAL gene was detected by Streptavidin-HRP (1:100, PerkinElmer, Belgium) followed by application of tyramide-biotin (1:25 in plus amplification buffer (PerkinElmer)) application. Then HRP (Horse Radish Peroxidase) was inactivated using 3 % hydrogen peroxide (20 min). After this slides were simultaneously incubated with streptavidin-Cy3 (1:100, Sigma-Aldrich)) and anti-digoxigenin-HRP (1:100, Roche) diluted in blocking buffer (Roche) followed by tyramide-biotin amplification. Biotin from the second layer was detected by Streptavidin-FITC (Vector Laboratories, Burlingame, CA).

Zeiss AxioImager M2 (400× and 1000× magnification) equipped with an AxioCam MRm camera and Zen software (Zeiss, Zaventem, Belgium) were used to analyse the hybridisation signal and to capture all images. Relative distance to the signal was calculated by the following formula:

Distance from telomere of the long arm to the signal × 100 % / length of whole arm.

Images were analyzed using ImageJ software. Signal position was measured using the Micromeasure software [51], version 3.3 (http://sites.biology.colostate.edu/MicroMeasure/). Pachytene chromosomes with a similar length were chosen for calculation of centromere index and percentage of heterochromatin. At least 9 pachytene chromosomes were used to determine the location of the Tyramide-FISH signals (Table 2).

**Table 2:** Characteristics of pachytene chromosome 4 and 7 of *R. wichurana*

Chr. Nr.	Chromosome length (μm)	Short arm (μm)	Long arm (μm)	Short arm heterochromatin (%)^a^	Long arm heterochromatin (%)^b^	Total heterochromatin (%)^c^	Centromere index	NOR	n^d^
4	45.6 ± 5.4	11.4 ± 1.4	34.0 ± 4.0	62.0 ± 6.0	21.0 ± 4.5	31.0 ± 4.0	25.0 ± 0.5		7
7	38.4 ± 4.2	4.0 ± 0.6	33.3 ± 3.5	88.0 ± 6.7	16.8 ± 4.4	24.3 ± 2.3	10.3 ± 0.8	+	9

^a^– calculated by the formula: length of heterochromatin of the short arm × 100%/total length of the short arm

^b^– calculated by the formula: length of heterochromatin of the long arm × 100%/total length of the long arm

^c^– calculated by the formula: (length of heterochromatin of the short arm + length of heterochromatin of the long arm) × 100 %/total length of the chromosome

^d^– Number of pachytenes used in the measurements of chromosome lengths and calculation of % heterochromatin

## Results

### High quality pachytene chromosome preparations suitable for Tyramide-FISH were obtained for Rosa wichurana

**Fig. 1:**
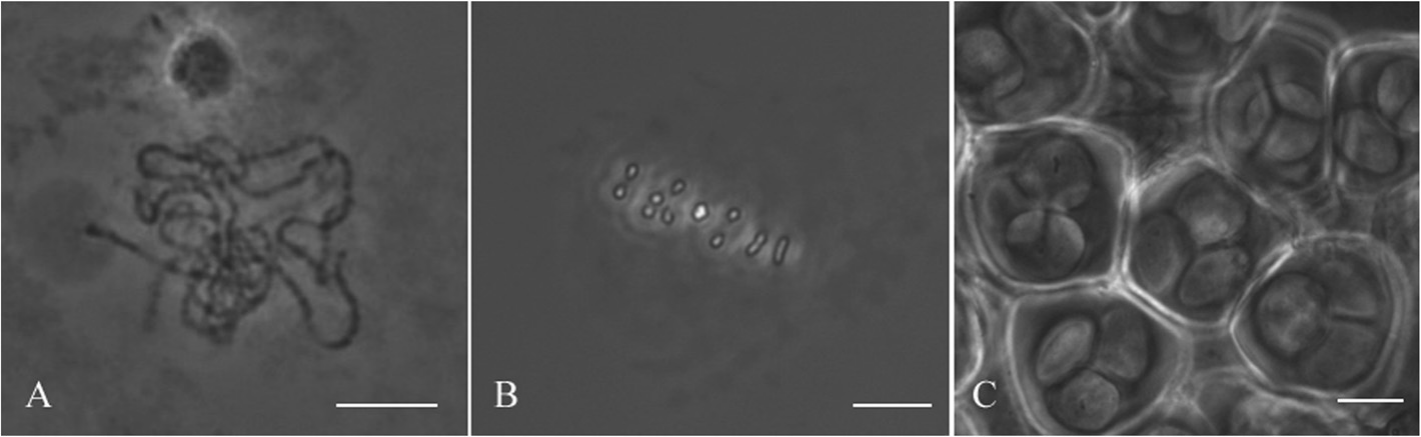
Pachytene (**a**), Metaphase I (**b**) and Tetrad (**c**) stages found in a *R. wichurana* flower bud. Bars = 10 μm

Flower buds with a hypanthium size of 3–5 mm had the most PMCs in pachytene stage although also PMC in metaphase I and tetrad stages could be observed in the same flower bud (Fig. 1).

**Fig. 2:**
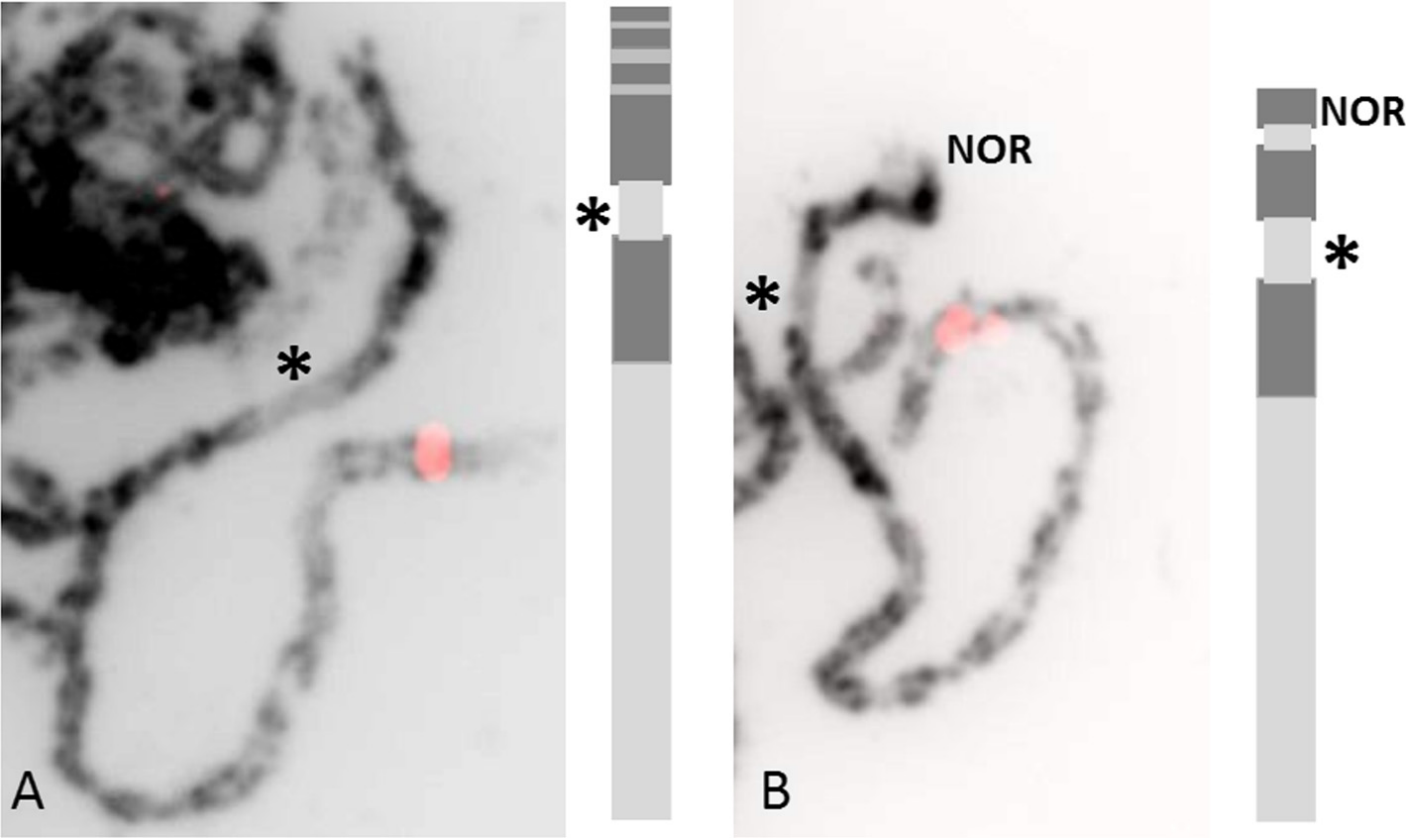
Inverted DAPI pictures and ideogram of chromosome 4 (**a**) and 7 (**b**). Red signals showed the location of AAA-2 (**a**) and MGM (**b**) genes after Tyramide-FISH to verify the correct chromosome number. Stars indicate the centromere position on the chromosomes

DAPI staining of *R wichurana* pachytene chromosomes did not reveal strong heterochromatin blocks that can be used as cytogenetic markers. All centromeres are the weakest stained parts of the chromosomes flanked by pericentromeric heterochromatin. Only chromosomes 4 and 7 are distinguishable by their clear presence of heterochromatin patterns on the short arms (Fig. 2, Table 2). In addition chromosome 7 possesses NOR (nucleolar organizing regions) on the short arm.

**Fig. 3:**
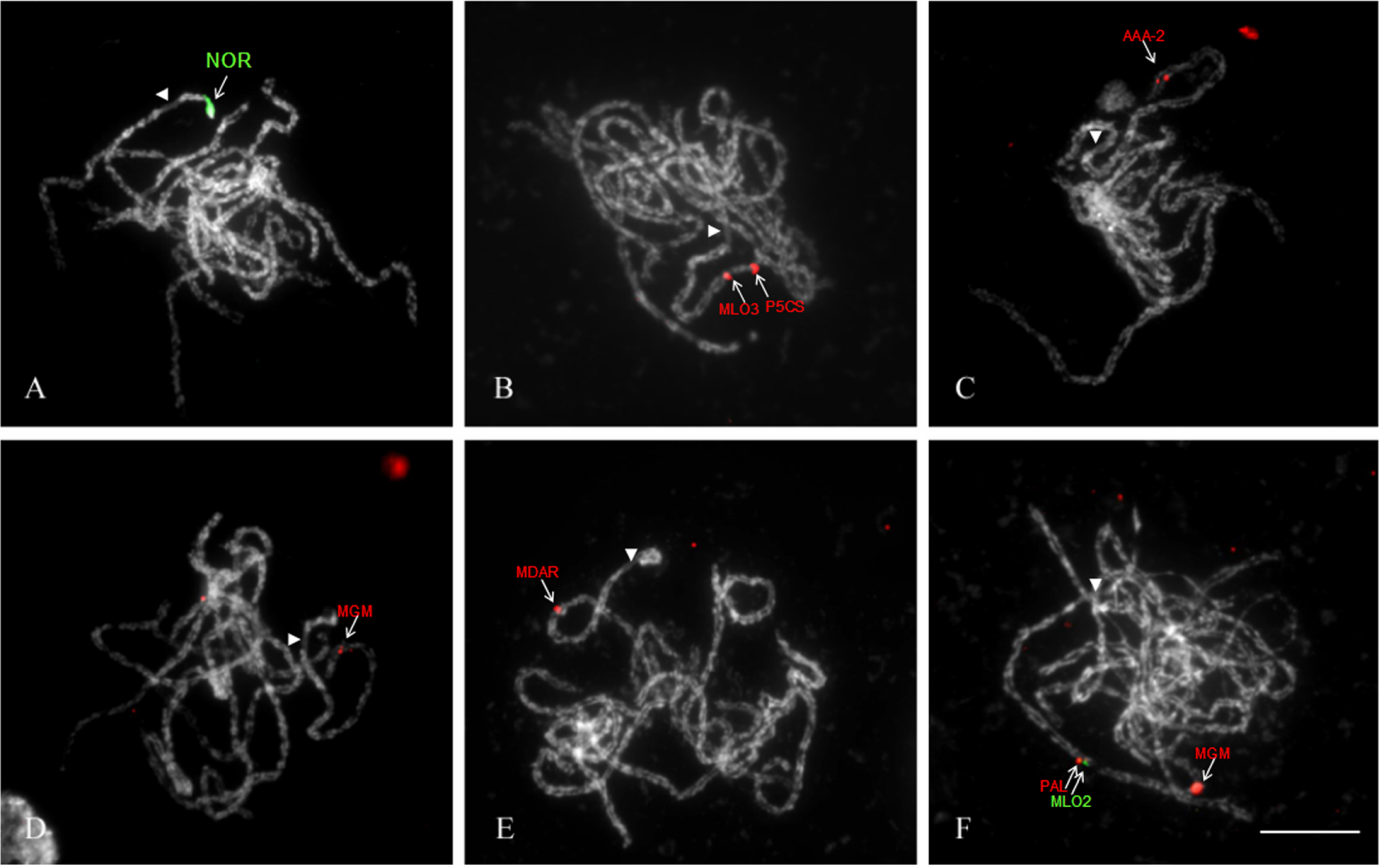
*In situ* physical mapping of genes on pachytene chromosomes of *Rosa wichurana*. FISH with Dig-labeled 45S rDNA (pTA71 plasmid) (**a**); Tyramide-FISH with MLO3 and P5CS genes, both labeled with biotin (**b**) and with AAA-2 gene, labeled with biotin (**c**); Tyramide-FISH with MGM gene labeled with biotin (**d**) and MDAR gene labeled with biotin (**e**); Sequential multicolor Tyramide-FISH with digoxigenin labeled MLO2 gene and biotin labeled PAL and MGM genes (**f**). Centromere of the chromosome that contains the physically mapped gene(s) is indicated by an arrowhead. Bar = 10 μm

To prepare pachytene chromosomes, the SteamDrop protocol was modified by adding a drop of 60 % acetic acid after the second steam application and before drying the cells. This modification improved the chromosome spreading and resulted in a clear 45S rDNA signal on chromosome 7 of *Rosa wichurana* (Fig. 3a). Moreover, application of Tyramide-FISH on pachytene chromosomes revealed differences in signal frequency between the slides prepared by 15, 30 and 60 s of acetic acid treatment on the heating plate (the final step in modified SteamDrop procedure). The maximum signal-to-noise ratio (visual observation) and signal frequency (60–70 %) were obtained on the slides prepared by 30 s of acetic acid treatment. Both 15 s and 60 s of acetic acid treatment resulted in low signal frequency (10–15 %). In addition the level of background was higher for 15 s and very low for 60 s.

### Pachytene chromosomes of Rosa wichurana provide up to 20 times higher resolution compared to mitotic chromosomes

The total pachytene chromosome length varied between 235 μm and 411 μm, which is 10–20 times longer than the mitotic chromosomes (20 μm ± 1 μm; [29]). The chromatin compactization of pachytene chromosomes of *R. wichurana* is 2.4 - 1.4 Mbp μm^−1^, calculated based on the genome size of 562 Mbp [52]. Taken into account the 0.2 μm - resolution limit for fluorescence microscopy, it can be concluded that the spatial resolution of FISH on *R. wichurana* pachytene chromosomes is between 300–500 kb.

### High resolution physical mapping of gene fragments using Tyramide-FISH

Seven gene fragments resulted in clear Tyramide-FISH twin signals on one pachytene bivalent (Fig. 3, Table 3). Only Tyramide-FISH for the gene RIN-2 resulted in multiple signals distributed over all the chromosomes.

**Table 3:** Physical location of the target genes on *R. wichurana* pachytene chromosomes

Gene name	Chromosome number/ arm	Location on chromosome arm (%)^a^
MLO3	4/L	44.0 ± 1.5
AAA-2	4/L	8.0 ± 1.0
P5CS	4/L	30.0 ± 2.0
MLO2	7/L	44.8 ± 0.5
MDAR	7/L	52.0 ± 1.5
MGM	7/L	18.0 ± 1.5
PAL	7/L	46.6 ± 1.0
RIN-2	Multiple signals	-

^a^- Distance from telomere of the long arm to the signal × 100 % / length of whole arm

Three genes were mapped on the long arm of pachytene chromosome 4. The AAA-2 gene was mapped in the distal part (relative distance of 8.0 ± 1.0 %) of chromosome 4 (Fig. 3c). The MLO3 and P5CS genes were mapped on more proximal positions, with a relative distance of 44.0 ± 1.5 % and 30.0 ± 2.0 %, respectively (Fig. 3b).

Four genes (MLO2, MDAR, MGM and PAL) were mapped on chromosome 7. MGM was located in the distal part of the chromosome (relative distance of 18.0 ± 1.5 %) (Fig. 3d). PAL, MDAR (Fig. 3e) and MLO2 genes were physically mapped in the middle of the long arm of the the pachytene chromosome 7 with relative positions of 46.6 ± 1.0 %, 52.0 ± 1.5 % and 44.8 ± 0.5 %, respectively. Tyramide-FISH with pairs of the genes (MGM + MLO2, MLO2 + PAL and PAL + MDAR) confirmed the order and location of all genes on the same chromosome.

### Tightly linked genes can be distinguished using high resolution multicolor Tyramide-FISH

**Fig. 4:**
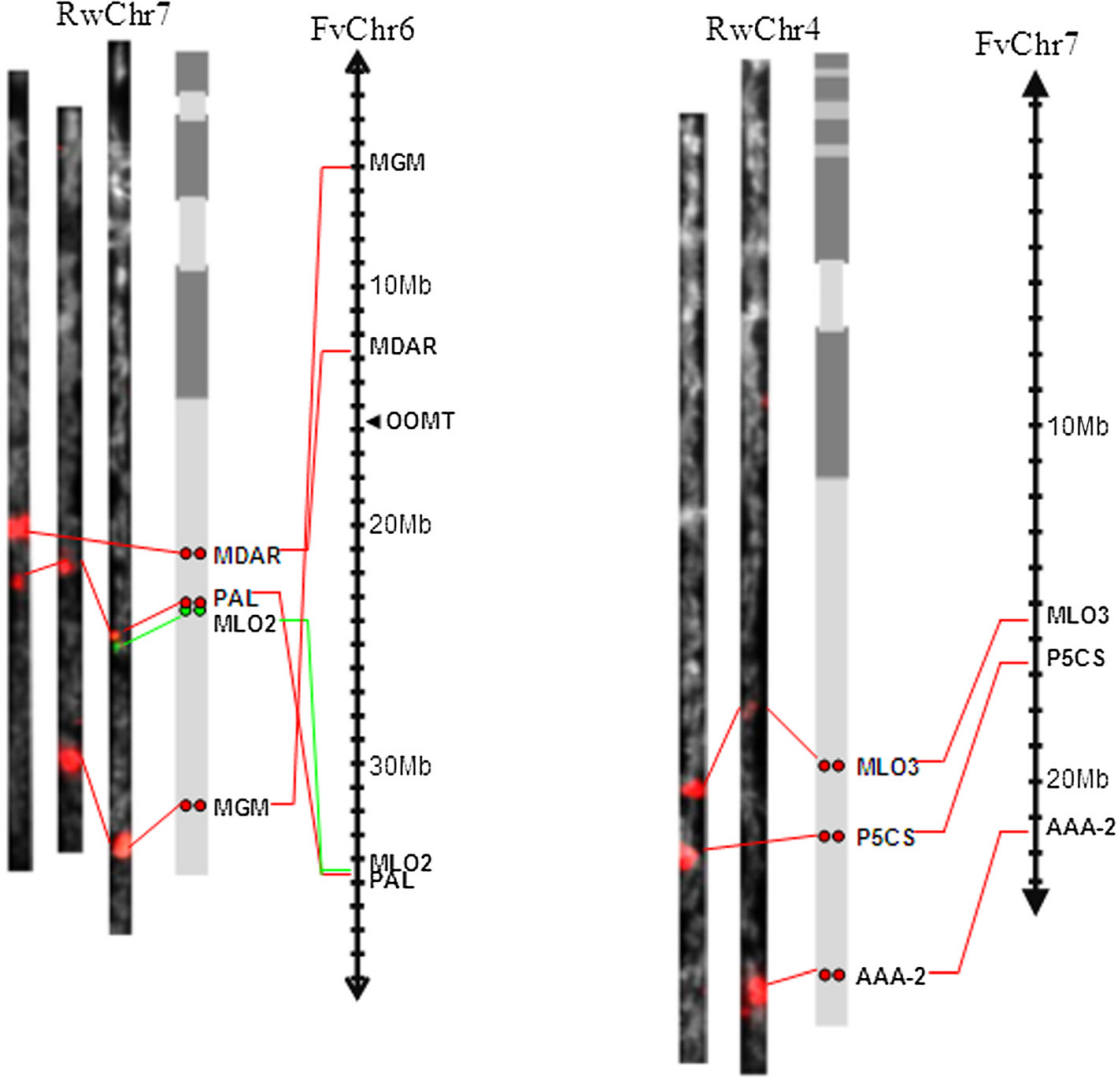
Physical location of 7 target genes on *R. wichurana* chromosome 7 (RwChr7, *left*) and 4 (RwChr4, *right*) versus *Fragaria vesca* pseudochromosomes 6 (FvChr6) and 7 (FvChr6), correspondently. Signals are shown on digitally straightened pachytene chromosomes and on an ideogram of *R. wichurana* and compared with pseudochromosome 6 (FvChr6) and 7 (FvChr7) of the *F. vesca* genome sequence

Because PAL and MLO2 are very closely located on chromosome 7, Tyramide-FISH with PAL and MLO2 resulted in one large signal. Therefore, to determine the order of PAL and MLO2 genes on chromosome 7, multicolor Tyramide-FISH was applied (Fig. 3f). Two-color Tyramide-FISH revealed the order of MLO2, PAL and MGM genes on the same chromosome. In 58 % of the observed pachytene cells (*n* = 17) closely located red (PAL) and green (MLO2) signals were observed, while the red and green signals in the other 42 % of pachytene cells partially overlapped. These results suggest that the distance between MLO2 and PAL genes is on the border of the spatial resolution of Tyramide-FISH on pachytene chromosome of *R. wichurana*. Based on the length of the pachytene bivalents carrying non-overlapped signals (41 ± 1.5 μm), the relative length of the mitotic chromosome 7 (10.0 ± 0.1 %; Kirov et al. [47]), the genome size for *R. wichurana* (562 Mb/1C, [52]) and 0.2 μm - resolution limit for light microscope, we may calculate the physical distance between the MLO2 and PAL genes which is about 270 kb. An overview of the genes located on chromosome 4 and chromosome 7 and their order is shown in Fig. 4.

## Discussion

### Chromosome preparation is a key factor for Tyramide-FISH success

To our knowledge, this is the first report on the application of FISH on pachytene chromosomes of a member of Rosaceae family. Pachytene chromosome preparation using the SteamDrop procedure has number of advantages [47]: 1) After cell suspensions are prepared they can be stored for months; 2) The chromosome preparation takes only 3–5 min from made cell suspensions; and 3) up to 20 slides can be prepared from one flower bud. Here we modified the SteamDrop protocol [47] for easy pachytene chromosome preparation of *Rosa wichurana*. One of the modifications is a final treatment of cells by acetic acid providing better chromosome spreading and Tyramide-FISH results. The time of treatment with acetic acid significantly influences the Tyramide-FISH results. Over-treatment as well as insufficient treatment resulted in a manifold reduction of signal frequency. PMCs require an optimal time of acetic acid treatment for reducing the amount of cytoplasm and the thickness of the organic layer covering the chromosomes [53]. Overtreatment of chromosomes by acetic acid results in histone elimination [54] and chromosome becomes flatten which reduces the chromatin accessibility [55] and the amount of electron-rich amino acids (e.g., tyrosine, tryptophan, phenylalanine) required for tyramide anchoring after HRP activation [56]. Therefore the chromosome preparation procedure is the most important step in high resolution physical mapping using Tyramide-FISH and should be optimized first to obtain satisfactory results.

### Resolution of FISH on pachytene chromosomes of R. wichurana

Mitotic chromosomes of *Rosa wichurana* are very small, ranging from 2.2 to 3.7 μm in length [29]. Their small size leads to a lower resolution (5–5.5 Mb) when using FISH, which hampers the use of FISH for physical mapping in *R. wichurana*. In contrast, pachytene chromosomes of *R. wichurana* are up to 20 times longer than mitotic chromosomes, which is comparable with data obtained on banana [41], *Arabidopsis* [39], tomato [57], *Medicago truncatula* [32] and rice [58]. Based on the genome size for *R. wichurana* (562 Mb/1C, [52]), 0.2 μm - resolution limit for light microscope and total length of pachytene chromosomes (235–411 μm) we can conclude that the spatial resolution of FISH mapping on *R. wichurana* pachytene chromosomes is 270–500 kb. Pachytene stage and location of probe in euchromatin or heterochromatin region influence on FISH resolution [14]. Resolution of FISH is much higher for zygotene, leptotene and early pachytene. For example the order of partially overlapped BAC clones can be determined on early pachytene chromosomes [35]. DNA condensation varies significantly along a pachytene chromosome – it is highly condensed in heterochromatin regions and less condensed in euchromatin regions [30, 59]. For example, the FISH resolution in the euchromatic regions of tomato pachytene is 10 times higher than in heterochromatin regions [30]. For *Rosa wichurana*, clear pericentromeric heterochromatin was observed on all pachytene chromosomes but also a number of weak stained heterochromatin bands were identified in euchromatic parts. Therefore, further study is necessary in order to estimate the precise resolution of FISH in heterochromatin and euchromatin of pachytene chromosomes of *R. wichurana* to convert microscopic distance into base pairs.

### Advantages and limitations of Tyramide-FISH for high-resolution physical mapping

Tyramide-FISH on pachytene chromosomes resulted in a higher signal frequency compared to mitotic chromosomes. We were able to visualize the signals on 70 % of the pachytene spreads. This is much higher than reported for Tyramide-FISH on mitotic chromosomes [29, 60]. Moreover, Tyramide-FISH allows physical mapping of short DNA fragments. Gene fragments with a length as small as 1.7Kb were successfully visualized on pachytene chromosomes of *R. wichurana*. Because most of the genes are free of repetitive DNA it is not necessary to use blocking DNA (e.g. *C*_*o*_*t* fraction) for physical mapping as is the case for physical mapping by BAC-FISH [61]. The efficiency of physical mapping using Tyramide-FISH is high. In this study 7 out of the 8 (87.5 %) isolated genes were successfully mapped on the pachytene chromosomes.

However, Tyramide-FISH have some disadvantages for physical mapping. Tyramide-FISH is highly dependent on the quality of the slide preparation. And another drawback of Tyramide-FISH is that sometimes gene fragments give multiple signals which cannot be reliably physically mapped, e.g., the RIN-2 probe in this study and [62]. In addition, multicolor Tyramide-FISH is a quite time-consuming process because each probe is detected sequentially.

### Advantages of an orthology-based probe design

Different strategies can be used for single gene probe design for FISH mapping. One approach is the use of EST [29] or genomic sequences [59, 62] of genes for primer design and further cloning of PCR products. Another strategy is to isolate orthologous gene fragments of one genus based on the whole genome sequence of another closely related genus. This approach is useful for the isolation of genes of which no full-length sequences or mRNA sequences are available in databases. This latter approach allowed for *Rosa wichurana* the design of specific primers and amplification of gene fragments with the predicted size based on the genome of *Fragaria vesca*. Moreover, it allowed the development of probes containing exon-intron fragments of the genes. On the contrary, EST clones often are too short as FISH probes and can contain highly conserved exon sequences which cross hybridize with other members of the same gene family, resulting in multiple signals ditributed along all chromosomes [62].

### Macro-synteny between R. wichurana chromosome 4 and 7 and Fragaria vesca pseudochromosomes

In our study, three genes, physically mapped on *R. wichurana* chromosome 4 (RwChr4), showed a perfect collinearity with *F. vesca* pseudochromosome 7 (FvChr7). The collinearity between *R. wichurana* chromosome 7 and FvChr6, however, is not well established yet. Tyramide-FISH results on mitotic chromosomes showed that OOMT and PAL genes, belonging to the same pseudochromosome FvChr6 of *F.vesca,* were located on two different chromosomes (chromosome 1 and 7) in *R. wichurana* [29]. Previously Gar et al. [48] also found that genes located on FvChr6 are located on two different linkage groups of *R. wichurana*, suggesting an ancient translocation event. Here we physically mapped 3 additional genes located terminal (MLO2 and MGM) and proximal (MDAR) on FvChr6. Our Tyramide-FISH results show that these 3 genes are all located on chromosome 7 of *R. wichurana.* Gathering all results from physical mapping on mitotic [29] and pachytene chromosomes and genetic mapping [48], it can be hypothesized that FvChr6 has a complex evolutionary history since *Fragaria* and *Rosa* were diverged from a common ancestor.

## Conclusion

Tyramide-FISH mapping of single-copy genes on pachytene chromosomes opens possibilities for the development of detailed physical maps of *R. wichurana* chromosomes. This approach will assist the integration of physical and genetic maps and will accelerate comparative genomic studies of genera in the Rosaceae family. For further experiments, cytogenetic marker development would be valuable for the identification of all pachytene chromosomes of *R. wichurana*. The application of larger numbers of single-copy gene probes covering all chromosomes of the karyotype of *Rosa* and the construction of the integrated genetic and physical maps for all chromosomes of *R. wichurana* is the scope of our future research.

### Availability of supporting data

The partial sequences of the gene fragments used for Tyramide-FISH are available as Additional file 1.

## Additional file

Additional file 1:
**The partial sequences of the clones used for Tyramide-FISH.**
