# Supplementary material for: High resolution physical mapping of single gene fragments on pachytene chromosome 4 and 7 of Rosa
Source: BMC Genet. 2015 Jul 2;16:74. doi: 10.1186/s12863-015-0233-9 (PMC4488978; doi:10.1186/s12863-015-0233-9)
Supplement: Additional file 1: — The partial sequences of the clones used for Tyramide-FISH. [file 12863_2015_233_MOESM1_ESM.doc]

>MLO3_F

AAAACACCAACATGGGCAGTTGCtGTGGTCTGTTTTGTGTTGCTTGTAATATCAATTTCCATTGAGCATGTCCTAAACTTCATAGGaAAGGTAAGAATAAAATGCTTGATCAATTGGTTTTTGATAATTATAATCTTCCAAGTTAATTGTTCATTGGAAAAGACATTGCAATGGAAAAAATTTCAGTGGTTTAAAAGTAGACAAAAGCGTGCTCTCTATGAAGCTCTTGAGAAGCTCAAATCAGGTATCGCATCTTGTGGGAACATTTAATTTTTTATTTTTTCCAAATACGAACTAGTTATGTCTGTTCTTCAAAGTCCTAACCGTTAATTTGTTTGTTCTATGTGAATTGTTTGAAGAGCTTATGCTGTTGGGATTCCTGTCCTTGCTCCTAACAGTTCTACAAGAACCAATCGCTGGTATATGTATACCGAAGAGTGTTGGAGCCACTTGGAATCCTTGCAATCATGGCACTTCAGATAGTAAGGCTAGAAAACTTCATGAATACTCAGATTCTGTCTTCAGTTTCCGGCGCAAATTAGCCAAAAAAGGAACTGATAAGTGCAAGGAGAAAGTAAGGGGAAGACGGTTCTTTTGTTATGCTGTTTCCATCATTGATACCCTACTCTGGCTATTTTCCTGTGTTCATGAATCTTAATCATGGGAATGTGAAGAAGTGTGTTCTATATTTGATGTAAGAATATGTGAATTGAAATTGAATAACAATCTAAATTGACTCTTTATTTCTGTAGGGTAAAGTTGCCTTTGTATCTTCATATGGGATTCACCAGCTCCATGTGTTTATCTTTGTGATAGCAGTTTTTCATGTGCTCTATTGCATCACAACCTTGGCTTTGGGAAGACACAAGTAAAGTGGCAAATGCGATTTTGAAATCTCTCTCTCTCTCTCTCTCTCTCTCTCTCTCTCTCTCTCTCT

>MLO3_R

AAAACACCAACATGGGCAGTTGCtGTGGTCTGTTTTGTGTTGCTTGTAATATCAATTTCCATTGAGCATGTCCTAAACTTCATAGGaAAGGTAAGAATAAAATGCTTGATCAATTGGTTTTTGATAATTATAATCTTCCAAGTTAATTGTTCATTGGAAAAGACATTGCAATGGAAAAAATTTCAGTGGTTTAAAAGTAGACAAAAGCGTGCTCTCTATGAAGCTCTTGAGAAGCTCAAATCAGGTATCGCATCTTGTGGGAACATTTAATTTTTTATTTTTTCCAAATACGAACTAGTTATGTCTGTTCTTCAAAGTCCTAACCGTTAATTTGTTTGTTCTATGTGAATTGTTTGAAGAGCTTATGCTGTTGGGATTCCTGTCCTTGCTCCTAACAGTTCTACAAGAACCAATCGCTGGTATATGTATACCGAAGAGTGTTGGAGCCACTTGGAATCCTTGCAATCATGGCACTTCAGATAGTAAGGCTAGAAAACTTCATGAATACTCAGATTCTGTCTTCAGTTTCCGGCGCAAATTAGCCAAAAAAGGAACTGATAAGTGCAAGGAGAAAGTAAGGGGAAGACGGTTCTTTTGTTATGCTGTTTCCATCATTGATACCCTACTCTGGCTATTTTCCTGTGTTCATGAATCTTAATCATGGGAATGTGAAGAAGTGTGTTCTATATTTGATGTAAGAATATGTGAATTGAAATTGAATAACAATCTAAATTGACTCTTTATTTCTGTAGGGTAAAGTTGCCTTTGTATCTTCATATGGGATTCACCAGCTCCATGTGTTTATCTTTGTGATAGCAGTTTTTCATGTGCTCTATTGCATCACAACCTTGGCTTTGGGAAGACACAAGTAAAGTGGCAAATGCGATTTTGAAATCTCTCTCTCTCTCTCTCTCTCTCTCTCTCTCTCTCTCTCTCT

>MLO2

TGGTCGGCTAGCATTTTTCTCAAATGAGAAATCCCTTGTGTGAATTTGGATTTCGTGTTGAATTCGATTACTATGACCAGGTAGAGGATTGTTCACCGAGTTAGGTTGATAGTGAGCACTAATCTCTTCCTCCTGACCCTGGTTAGACCACGGTACATCATCATTGTCGTGGCGAGATGGGGACTCGTTTGGACCTTCACTTTCAAAGTACGACATCCTTGGCAATGTATGCATACTTTCCACGTCCTGTTCGGATTGATAATGCCGCAGAAGATGAACAGGGGACATGCCATGTAATGGAGTGCCGGGTGCACTGGTTGGAACTGAATGCTTGCTCTGTTTTACATGCTTCTTTGCTGCATGGTGCCACTTTCTTATAGCATTTGCCACTCTATCATTAAAAATTGTAGGCTTCATAGTTGAACCCATCTGCATATAAAGTGTGAATTTCTTTAGTTCACATTTCTTACTGTGCAAGAAACAAAGTATTGAGTAAAAATCAGTCTGACCTGTGTTACCAAGGCATAAAGAGGAAGAGTTACATAGCTGCATAGTATTTGTATGATGGCCCTGCTCAAATCACATAAATAAATTGACTCAGTAAATAAAGTCCTTCATAATCAATAATGTCTTTGTAATTAAGCTATAAAGTTAGAAAAATAACTAACCCCATTGTTATTCTTAAGACTACATCTTCCAACTTGTCATGGAAGCAAGACTTCAACCCAAATTCATACTGTAAAAGAAGAGAAGAGAAATGATCAACTTTGAGATTTTTGCAAGATGAAAGGTAAAACCATCTTGGCTAATGAGAGTGACAATTTGATGTGAGTTTACCCAAGTCCACGCAAAGAAAGCAAGTTGAAAAGCATT

CTGTGAGGAT

>AAA2_F

GTTCCCTTTGTMTTGCAGATGCAACAACATTAACTCAGGAAAGTTTTGCTCCTCTTTTATTATTTTTCTGTAATTGAATGTCATTTATGCAGAGTATTATTCAGCCTGTATACTCTGTTGCACACAATGCTGGCCTTCATGATCCRAAGGAAATAATTAACATTACACGCCTATTTTTCTCAAATGGTTTTTGTCTCAAATTCTGCTGCTATGCATATGACAATGAAAACAGAGTTAGTAGCGATTCCATATGCATTTTTTCTGTCATTCTGATATGGCCATGAAATCATTTCGTCACATGCAGGCAGGATATGTGGGGGAAGATGTGGAATCGATTTTATACAAACTTCTTTCGGTATGTATTCACTAGCATATTCTATTCACGTCTAGCTAAATTTTTTATATGTGTTCAGTCTATTATGGAAGCATTTTTTGTACCTTCGATTTATTCTTGTAACTGCGTCCTTCTATGCTATTTACCTTTTATTAAAATCTGTGAGAAAAGTAAAGCTGTTTGAATTGTCTATGCCATAATTCTCATATGATAATTGTTTGCATCAAAGCTCCTTTGTCCTCTGTTACCGTTTTGATTTAAAATGACTTACTGTTTTATCATCAAGAAAATACTTTGGAGTCTACACTTCTATACTTATTATGGACTCAGATCATGTATCTTTATCTGTATTCTATGCCAGGTTGCTGATTACAATGTGGCAGCTGCACAACAAGGAATTGTTTATATTGATGAAGTTGACAAGATTACCAAAAAGGTTCATTTACACCCTCTCTCTCTCTCTCTCTCTCTATCTCTATCTCTATCCCCCACCTCTCTCTCTCTCTCTCTCTCTCTCTATCCAGTTTMCCACTCCTTTATATCTGCCTACATTAGCTCCTAT

>AAA2_R

ACGGCCTCTTCATCAAKTACAACTGCATCTATTATGTCAKCACCAGTTCTAACATCAGGAATCTGSRAAAGAATGAAGAATCCATTAGCTATTATKTCCTGTTTGGAACCAAATAAATAATTTRCTTGGTTCATCTGTRRTCTGCTTCGGGCASAACCACARAWCCTCACTTGGTTTGTAAATGAGTTCATAKGTTGGGRACAGAAAATTATATTATGCAATTTGATGCACAWCATTTCCTAAATTACTCATTATACCATTCTTCARGAATGGTGTCGATCCTACATAGGACCACCTCTTCAAGTAAATGTTCATTCACATATCCTCRGGAAGAATTTTGAAATGAACTCTAATATGACATTGKMAATCCTTKACAATTGAGGAAATTATTAGGTTCCAATCACTCCCTAACTGARAAATCACATTGGTTCCAATCACATTGCTTTACTGATACTTGTGGATACAACCAACAAGCCACTGAGTTAAAAAACTTGGTTCAAGTACTTGGCTTCTGAAACCARGCACCCAATTAATATGTMGGCTTCTTCTAAATGCTGATTTGCACAAAGTGAGCAAGACTCTAATCCTTAAGATGGGGCAAATCGCAAATTAGTACTTCRAGAC

ATTTCCCCTTCCCCAGATWTCRAGATAATTGTGCTGAGAGAAAAATTAGTCCCAGCATTMAGGTATTCACCCCAATCCMTTCCAAGTTATTAACATGTTCAGATTGCAACAGAAAAGATAAGTGGCCTTACCTCATACATAGCATCCATTAATATAGTTTCTAAAAGTGCTCGTAATCCACGAGCCCCAGTGTTCTTTGATATTGCTTTCTTAGCMAMTTAGCCTCAAGGCACTTTCTGTGAAATGCAGTTGGACCTGAGARAAGTAAATCATAAAATATAGGTACAACAGAAGCATGCTTGCAAAGGAAGTGAGGAAACAAARATTGCATTCAACATGCAMACAGCAACGTATTCTTCACGAAAAWAGCAGCCAAACAAGAATGCTACGTCATCATTCGGAACATCTTCTGATTGCTCCCCAATGCATTTTTGGACTCGTAGACTACGAACATCTTGCAGTAGAAAAACAAAATGGGAATGKTAMCTTAAAAAWGAT

>MDAR_F

AAACTTGGGCTTTGGTGAGGTTGGATTGTTGTCTCCAAAGAGCACGGTCTCACCAACATTATCGCCATAGAATTGCCATGACAGATCAAAGGAGCGAGAATAGAAGAATGGAAGGTAATCATAGACCTCAACTGTCTTTCCCTCCTCGCTTGCCTTGATGGCCTGCAAAATCAAGACACTATATGATTCCTGGATCTTTGGGAAGAATTTTAGAAAATCGGGTTCCAACTCATTTGCCAGAATAATGGAAATGAATTCACAGTGGACATAAATAAAGATCTAAGGGGAGCTGCATACCTTACGGC

CTGCTCTGCAGATTTGCGTGCACGGTCAACATGCTCGACTCTTCTGTCCTCATTGTACAACTTCAAAGGGAAAGTGGCGACATCACCCACAGCATATACATCAGGAACACTTGTTTTAAAGAATGTATCCGTCTGCAATCAATAGCATATATCAAAATGGATTCCATGAATATTTAATTACATAATTAGGATTGACTAGTCCAATGTTAGAGATAAAAAGAAATACACTAAATATTGTAATGAAAATTGCTGTTACTTCAGAAAATCTGTACAGCCCACAATCAACAGCATATATCAAAATGGATTCCATGAATATTTAATTACAGAATTAGGATTGACTAGTCCAATGTTAGAGATAAAAAGAAATACACTAAATATTGTAATGAAAATTGCTGTTACTTCAGAAAATCTGTACAGCCCATAAAGACATCAAGTAAATTGTTTTGGTGTCCAACTTCCTTAATAACTCAAATGCAGTGTCCTTTCTAAGTAGCTTTCTTATTATGCGGTAAGATTGAATGCTCAATTGTATTGAAATGAATGATMAATCATTATTTAAG

ATAAACACTGTTATTAAAAACTTTAAGCATTAAAATAATTTACTTAAGTACGCAAAGGAGAGAATCAAGAAATATARAGTGACGATACTATCCACCTTCTCCTYTTCACTGTCCTGAACAGATGTAGGGCCTCCCGAMCACACACATGTCAGCTTAGCCAGTGTCATCCATTGAATGCACTTCTTGAACTCTGCATTW

>MDAR_R

GAGGCGGTATGGTTAATTTCACTTTGTTTATATCTAGTTGATTGTGGATTTCGAGATATTTATGCCATTAGTTGTTAAGTTTTTGACGAGTACATCAGAAATGGTTGAAGATATAACTGCCTACTTTTTACATAATTGAAGATATAACTTTCATCATTCATGTGGATGCCGTGCTTACTGATTATAGACTATAGTCACTGTACTTTTTATTATAGCGACTGTTTCAAATCTTTTTCTTTATAATGGCTGTTGGTATTTGGTTTTGTTTAATTATGAATTGTTAACTTCTCCTGTCTTTTTCTTGCAGGTTGCTCCCTATGAACGTCCTGCACTCAGCAAGGCTTATCTCTACCCTGAGTGTAAGTTGTGTTCTTTTCAGTTCCAACTTTCCTCAAAGCTACAGGAATTATTATGACACGTGGTTCTTACTTACACCTGGCACATCAGTGTAGATCAGTCTCCTTTTTGTATGTTTATCGCATTCAATGACATTGTAACTTAAAGTTATAATTGGTTTTTGCAGCTCCTGCTAGACTTCCAGGGTTTCATACGTGTGTTGGAAGTGGAGGGGAGAGATTGCTTCCTGATTGGTACAAAGAGAAAGGTTTGTTTCTCCTCTACATCACAGTTTATACCAAACTTAGTTACATAATTCCGGAGAGCTGTCCTAAGCAATTTTTGGAGATGGTTAAACAGACCACCATTGCTCATTCTTGTGCAAGAGTGCCAGACTCTATTTTTACTATGTTATTGAATGTTTCAATTTGATCTTTACTATGAACTATTCTGATGGAAACATTTTTTATAATTACAGGAATAGAGTTGATTCTGAGCACTGAAATCGTCAAAGTTGATCTTGCTGGGAAGACTCTTGTTAGTGGAACTGGGAATCCTTCAAGTTTCAGATTCTTATCATTGCAACTGGTTCAACTGTAAGCAACTCTACAAAATAATGGGAAGACTCTTTTATATTTAGCCATCTTTGCATAATGATAAGTGCATTTTTCTATTTATGTAGGTGTAAGATGTCGGATTTGGTGGTGAAGGAGCTGATGCTAAAACATTTCTACTGAAGAAATTGATGATGCTGATAGCTCAATGATGCAATTAAAGTCCAAAGATATTTG

>MGM_F

GAACAAAGGGATCTGCCATGCTTCTGGAAGCATTGTCGCTCTGATGGTAGCTGCAACAGGCATACCTACTGAACCAACCTCAGGATTGAACCCATATTTGTAAAAATCATCCTTAAAGAAGTCTTCAGGATTTTGGATTTCATAAGGGCCGTCAGTGTAGCCCCCCTTTCCATTTGCAAATCCATCCCACATTGATCCTTGTATGTAAATGCGTGTACCATCCAGATATTGGCTTGGATCCTTTGAC

ACTGACTTCAAATTTTCAACATACTCACCACTACCATTTATGGAATTTTCAAAATGTGGATGGAGCCTGAGGTCCTGTTTCAAAGCTTTGTTGATGTCATCGGGTGGGATTTGTTCATTTCCTCCCACCCAAAGAGCGAGACCTGGATGGTTCCTTAGAAGCTTGACAGTATCTCTTGCACATAGCAAGAAAAGATCATGGTCCAGTGGACCGTTTGGATTTGATACCGGGACACCTCGTCCATCTACATCTCCAGTAATCCAAAATTCTTGCCAGACCTAAAAAATTTAAATCAGAAGCAGTATGCATCAGATTCACCCAACTAATTCCATGGCCCATAGATCTCTAAACCTGTGCATTTAATTTTGACAACCAAAAACCTAACAGAGTAACAAGTCTTCTTAGACTCCTTCCCAAGAGATGCGCACTGAAAGAAAGAGCAAAGATAGAGGGAGAGAGAAGGATAGCTTACCAACAGGCCATAAATATCACAGTAATAATAAAATTCTGGCCTCTCAGCCAGTCCACCACCCCAACATCGGATCATGTTWAAATTCATA

TCTGCATGAAACTTGATGTCTGTTTTGTAACGCTTTTCTGAAAGCCGTAGGAGGCCATCTGGCAATATCCAATTACCACCACGAATGAAAATAGGCTGACCATTGACCTTGACACCCTGAAGAGTAGAACGTACAGGTCACACAAAACATAATATCCWACMCATATWTAGTCCTAGTGTATACCTTCCACGGTGGCATCATCAATGTGCTCTCGATTTGCGATCCAATAATTGCTCACAA

TCAGACYCTCATATCCTTATCACAGTATAACAACATGATCAGGCTTGCTTTCCATACCAATTTTGGACA

>MGM_R

CGGCATGGAAAATGAGTCAATCATAGATATTGCTGATTCAGGCAGGCWGTGGTWCACATTTTGGTACTTTACAACCTTTCAGTGTAAGCTGGTAAGTGATGCATATAAATGAAAAGAATGCACTTTGAGGTTGTGTTGAGGTTATTTCTCAATAGTTTGAATGATTACTGTCCATGTTAATCTTTACTTTCCTTGGCATTGTGGTTAGGAATAATGTTGTCTATMCACTTGGCTCTTTTACAGTCAGGTACTCAGCACCTGGATCTGAATTTCCGTGGAATCAATTACTATGCARAGGTATATTTGAATGGGCACAAAAAGGACATCCCAAGAGGGATGTTTCAAAGGCATTCTCTAGATGTCACTGATATAGTACGTGACGGTGAAAATTTGCTTGCTGTTGTCGTTTACCCCCCAGATCATCCTGGGAGTATTCCTCCTCAGGGTGGGCAAGGTGGTGATCATGAGGTACATATCTACAATCCAATAAAGCTCATTGATTATATACATTCCACTGCATTCTCGGTTACAGTTTTTACTCACCAATTTCTAGTTTTCAGAACTACCATAAAGTGCTGGATCTCGCATGATTACTTTAAACAAATTAATTTTTTATGCAACTTGCC

AATGTGCAAACTAAGGTCATTAATTATTTGAATTTTAATTTCAGATTGGGAAAGATSTSACCACACAATACGTGCAGGGTTGGGATTGGATGTGTCCTATAAGGTGATTATGTTTTACTAGTGAGAATGCACCCTGTTTCGTTTTGTATTTTCTGGTCATATGGTGCTKTTCATCTTCCTTCAGTAAGGAATTCCAGAAAACTTGTCGGCGGATTTATTTTTATGTCAGCTAAAATGAGACCAAAGACTATTGATTGATGTCCGGGAGGAACTGCAATGTAATTGAGTTGTGATGCTGATAGATACAGCAATTTCCMGTGTTWTCTTGTGTGTATGTCTAGATTTTTTTCTTCYTCTKTGCTTGTACAGTGCAATCTTMGAACTTGWTTWWTTGAGATAGCAGCCMAAAAATCTCATTCCATTGAATTGTATCGTACAGATGGAACACCCGSCTATATGG

>PAL

AACCTCCCACAGACGAMCCAAACAAGGCGCTGCTCTTCAGAAAGAGCTTATTAGGTAAGTTACGTTGAAAATGTTTGCTTCGTCACTTTAACATGTTACGTTCTCAAAAAATTAATTTAGTCTGTAAAACTTACTATATCCTAACAACATCACTTATTAATTTTTTGATGGTATAATACATGAGTTACTGTTTGACAAAATAATTTATCTTCTATGGACTAATTAAGGGTCACTTTAGTATATTTAAAAATTAATTCTTCCATTGATATGTAAGCAAGATAGACAATTTACTCATTAATGTACATGGACCTGATTAAAACTGAGTCGATCAAGTCTCAACCTAGTTTCTTGTGGACCTGGGGAGGAAAGTTTCGCATAGGGAAAGCTTTATACCCCGGGATTTTTTGTCACTAGCTGAAATAGACAGTTCCTTGTCACCTAATATCTGTCTCGAAACCAACTTGGATTGCATAGGGCGGCAAACTTAGTACACGCACGTCATGAGTAGTAGTACTTGGAGGTTCAAGGAATTTACCAGTACAATCAATTGGGATTTC

CAACACCTAGTTAAATGGAATTTGATCAGATCTGGGTTTCTAGTTAATTAAACTGGTCCTTTTGGATTTGAATCCATTAATTTCTATCAATTTTATGTGGAATCATACGCATTAATGCATTTGGTTTTTAATATATTCTTTTCTGGATCCTCCGAATTATGAACTGAGCTTTTTCGCTTCTTGGTCTAGATTCTTGAATGCTGGAGTATTTGGCAACGGAACAGAGTCTCACACCCTGCC

TCACTCTGCAACAAGAGCAGCCATGCTCGTTAGAATCAACACTCTCCTGCAAGGCTACTCAGGCATAAGATTCGAAATCTTAGAAGCCATCACCAAGTTTCTCAACCACAATATTACTCCCTGCTTGCCTCTCCGSCGGCACGATCACCCGCCTCCGGAGACCTTGTGCCSCTGTCCTA

>P5CS

CTTCGGATCGCTAATGAAGCTATCTATTCAAATCATGCCAAAGAAAAGAAAAACAAAACT

ATATTTAGAAAGAATAGCAAATGCAACTGTTTGGAAAAACATACAATACATTAGAAACAC

ATGATATTAGCTTCTTTACAATTTTCCATGTAAATAACAGCATAAATTATCTTTTACTGT

CCAAAGTAAAGACTTGAATAAGATGTTACTTCCCAGTCCTTAGATTCCATGTACTGAGAT

GGATTTCAGCCATTTTTGCATCTCAGTCATCAATATTTGAACTAACAACATGCAGTTTGA

TATTCAGAAATTGTTACTTACCTGTACTAGTGCATCAGGACGGGATTCAAATACAATCAG

GAGAACACCCAATGGACTTGATGTTTTCTCTAAGACAAGACCATCTGCAAGCTGGAAATA

AGAGTAGTCAACAGAATCATTAGCAATTCATTAACAGAAGAAAACCAAGTTATGGTGCTC

TAGTTGGCAAAACATAATTGGTTACAACCAGATTACAGGCTATTTAACACAGATTCCACA

TGTCAATGACCATCATTATCTGTGTGTTAGTTGTTCTGTCAGATTACCATAAGCCGTAAT

TGATGATCCTTATACCCCTCCTTACCTCAGTCTTTTTCAAAACATGACCAATAGGATCTT

CCATGTTTGCAAGCACGCGAACAGAATTTGCAAGACTTGTAATCTGTCAAGTATAAAAAA

GCAGATTCACGAACATAAGTAGGAGTATGGTCGAGCAAGAACATTATACGCAGCATCAAA

CAGAAACATATTTTGGTGTGAAGTAACTTGGCGAAGTTGGTTTGCTTTACCTTGCCAGAC

TTTAGAGCCAGGCGAGATATCAAGGATTTTTCATATCCCGCTTGTTGTGCAGCAGAAACA

TCAGCTTCATTTTCAACAATGATCTTATTAGCATTTGCTTCAATGGCATCGGCTATATCC

AGAAGAATTTTTTTTCCCTTTGCTCTGAAGTCATGGGCCTTGTACAGAAGTATTAAACAG

TAGTATGCTGCTGAACTAGCCGATTTATTGCTAATAAGTTATGTGGACAAGGAGTGCTGG

TACTGCGAAAGAAAACCTATGAATCAACAAACATATCTCAATTTTTGGGGAT
